# Brain insulin sensitivity is linked to body fat distribution—the positron emission tomography perspective

**DOI:** 10.1007/s00259-020-05064-7

**Published:** 2020-10-07

**Authors:** Eleni Rebelos, Lauri Nummenmaa, Prince Dadson, Aino Latva-Rasku, Pirjo Nuutila

**Affiliations:** Department of Endocrinology, Turku PET Centre, Turku University Hospital, University of Turku, Turku, Finland

Dear Sir,

In a recent study by Kullmann and colleagues [1], the authors showed that brain insulin sensitivity predicts weight loss at follow-up of nearly 10 years. Furthermore, in a cross-sectional experiment, good hypothalamic insulin responsiveness was associated with lesser visceral fat mass, while no correlation was detected with subcutaneous fat mass. However, origins and mechanisms of brain insulin resistance remain elusive [2].

Whereas definition of systemic insulin resistance is based on tissue-level studies where several molecular defects have been established [3, 4], characterization of human brain metabolism relies on different in vivo neuroimaging methods such as positron emission tomography (PET), hemodynamic imaging (functional magnetic resonance imaging, fMRI), and neuromagnetic measures (magnetoencephalography, MEG). Accordingly, definition of brain insulin resistance varies depending on the implemented method: i.e., a blunted cerebrocortical insulin effect when MEG is applied [5], decreased intranasal insulin-induced suppression of hypothalamic blood flow in fMRI [1], and an insulin-induced increase in brain glucose uptake (BGU) in [^18^F]-fluorodeoxyglucose PET ([^18^F]-FDG-PET) studies [6]. Unfortunately, multiple measures are rarely used within a single study, and in the absence of a ground truth measurement of brain insulin resistance, the integration of these results is complicated.

It would thus be important to assess the consistency of these measures (MEG, fMRI, and [^18^F]-FDG-PET), when applied to the same pool of subjects. Second, complementary information within the same subjects would be acquired, since each of these methods characterize different aspects of brain function: MEG measures net effect of the ionic currents in neurons during synaptic transmission, BOLD fMRI measures combination of cerebral blood flow, and cerebral metabolic rate of oxygen, while [^18^F]-FDG-PET quantifies brain glucose uptake at the whole-brain level and also in specific parts of the organ [7]. Furthermore, these three techniques target at least to some extent different cell types. MEG picks up the neuronal ionic currents, whereas the [^18^F]-FDG signal in brain PET studies is likely driven by astrocytes [8], and fMRI presumably measures a combination of both.

We have previously reported in two different datasets with subjects undergoing bariatric surgery that increased brain substrate uptake (glucose or free fatty acids) before surgery predicts a worse glycemic control at follow-up [6, 9]. This finding is in line with the longitudinal findings of Kullmann and colleagues: brain insulin resistance and/or higher brain substrate uptake at baseline predicts an unfavorable metabolic outcome at follow-up.

Further, here we demonstrate, using [^18^F]-FDG-PET, that insulin-induced change in brain glucose uptake (BGU) correlates with visceral but not with subcutaneous fat mass. Data from 44 subjects (11 male/33 female, age 45 years [SD 10], BMI 35 kg/m^2^ [interquartile range 8]), who were studied with [^18^F]-FDG-PET during fasting and euglycemic hyperinsulinemic clamp with insulin infusion at 40 mU m^−2^ min^−1^ were analyzed. BGU was quantified using dynamic PET imaging, and abdominal visceral and subcutaneous fat mass were measured with MRI as detailed in the original publications [10, 11]. Prior to inclusion, each participant gave written informed consent. The studies were approved by the Ethics Committee of the Hospital District of Southwest Finland and conducted in accordance with the Declaration of Helsinki.

**Fig. 1:**
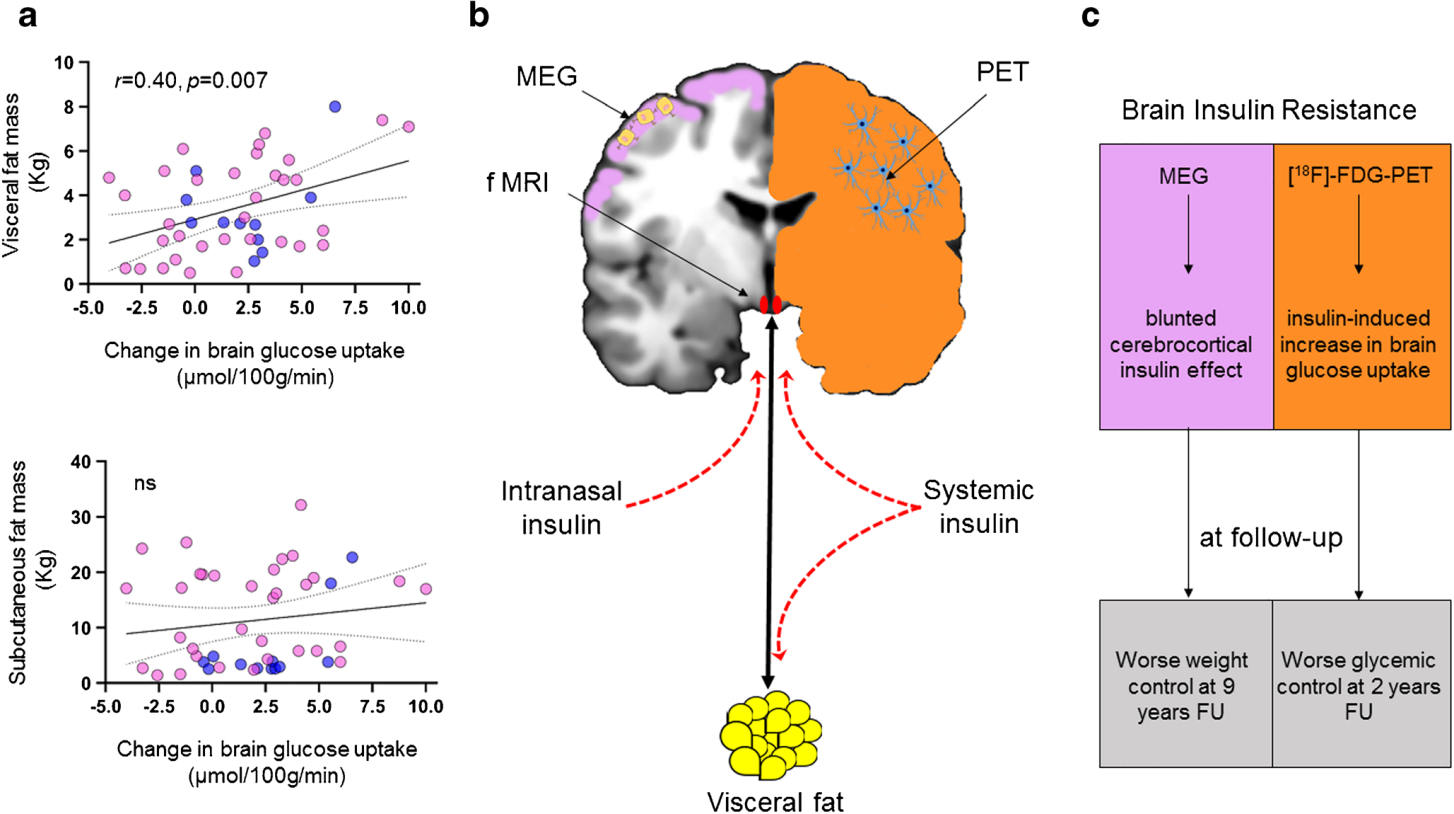
Change in brain glucose uptake between euglycemic hyperinsulinemic clamp and fasting conditions correlated positively with visceral fat mass, but not with abdominal subcutaneous fat mass. Pink circles are female participants, and blue circles are males (**a**). The data of Kullmann et al. and ours show that brain insulin resistance defined as smaller hypothalamic responsiveness to intranasal insulin or as increased BGU during hyperinsulinemic euglycemic clamp relates with visceral fat mass. Moreover, brain insulin resistance assessed with MEG and PET predicts worse metabolic outcome at follow-up (FU). Taken together, brain insulin resistance seems to be involved in the pathogenesis of systemic insulin resistance (**b**, **c**). MEG can address cortical areas (pink color), BOLD fMRI can detect perfusion changes in response to stimuli in very small brain areas, like the hypothalamus (red), while PET measures metabolism at the whole-brain or larger regional levels (orange) (the cartoons inside the brain represent neurons and astrocytes) **(b)**

We found (Fig. 1) that change in BGU from fasting to insulin-stimulated state correlated positively with visceral fat mass (*r* = 0.40, *p* = 0.007), whereas there was no association between increased BGU and subcutaneous abdominal fat mass, confirming thus the finding of Kullmann and colleagues [1]. We however found that the cross-talk between brain and visceral fat occurred in conditions of systemic hyperinsulinemia, and thus the activation of the brain-visceral fat axis could arise either through a direct effect of insulin in the brain, or in the visceral fat, or via bidirectional coupling. As for the differences between subcutaneous and visceral fat, it has been previously shown that the autonomic innervation differs in these two fat depots [12], and a previous study has shown that central insulin action through intranasal insulin administration suppresses systemic but not subcutaneous lipolysis in humans [13], suggesting that the visceral and subcutaneous depots are differently affected by central insulin action, a pattern which may explain also the present findings.

Based on the consistency of our findings and those reported by Kullmann et al., it is likely that all three definitions for brain insulin resistance may functionally converge, with insulin resistance in the brain manifesting as both impaired regional neuronal responsiveness to insulin and globally altered tissue metabolism. Furthermore, these studies using different methods indicate that visceral adiposity is linked to brain insulin resistance. Although findings from one neuroimaging method might eventually be translated into the findings of another, more studies combining different approaches on same study subjects are warranted to establish the relationship between different features of brain insulin resistance and their functional consequences.
